# Assessment of Nutritional Status by Bioelectrical Impedance in Adult Patients with Celiac Disease: A Prospective Single-Center Study

**DOI:** 10.3390/nu15122686

**Published:** 2023-06-09

**Authors:** Daria Maniero, Greta Lorenzon, Ilaria Marsilio, Anna D’Odorico, Edoardo Vincenzo Savarino, Fabiana Zingone

**Affiliations:** 1 Department of Surgery, Oncology, Gastroenterology, University of Padua, 35128 Padua, Italy; dariamaniero@gmail.com (D.M.); ilaria.marsilio@gmail.com (I.M.); edoardo.savarino@unipd.it (E.V.S.); 2 Gastroenterology Unit, Azienda Ospedale-Università Padova, 35128 Padua, Italy; gretalorenzon90@gmail.com (G.L.); anna.dodorico@aopd.veneto.it (A.D.)

**Keywords:** BMI, nutritional status, bioelectrical impedance analysis, gluten-free diet, celiac disease

## Abstract

The gluten-free diet [GFD] has been linked to an increased risk of weight gain and the development of metabolic disorders. Most of the studies have focused on the effect of GFD on the Body Mass Index [BMI]. We aimed to evaluate the nutritional status using specific nutritional parameters in patients with celiac disease [CeD] at diagnosis and on a GFD compared to healthy controls. We recruited subjects at our outpatient clinic at the University of Padua. We collected demographic and clinical data and values obtained with bioelectrical impedance analysis. A total of 24 CeD patients and 28 healthy controls were enrolled. CeD patients at diagnosis had a lower body cell mass index [BCMI, *p* = 0.006], fat-free mass index [FFMI, *p* = 0.02], appendicular skeletal muscle index [ASMI, *p* = 0.02], and phase angle [PA] [*p* < 0.001] compared to controls. Their percentage of extracellular water [ECW] was also higher [*p* < 0.001]. Considering CeD patients after GFD, nutritional status significantly improved after 6 months of GFD. We did not observe differences in BMI among groups [*p* = ns]. CeD patients at diagnosis were found to have a poorer nutritional status than healthy controls, with a positive effect of the GFD on their nutritional status, underlining the inefficacy of evaluating this aspect through only BMI evaluation.

## 1. Introduction

Celiac disease [CeD] is a chronic autoimmune disorder caused by the ingestion of gluten in genetically predisposed individuals [1]. CeD occurs in about 1% of the general population. It has a higher prevalence in females [2,3,4], with an increasing rate of diagnosis year by year, due to environmental factors and an improvement in disease knowledge [5,6].

In recent decades, CeD’s most common clinical presentation changed from classical presentation, with gastrointestinal symptoms such as diarrhea, malnutrition, and weight loss, to non-classical complaints or signs such as fatigue, iron deficiency, extraintestinal manifestations, or asymptomatic presentation, leading to problem of diagnostic delay [7,8].

Nowadays, the mainstay treatment for CeD remains a strict gluten-free diet [GFD] that permits intestinal damage recovery, proper absorption of nutrients, and symptom resolution in most cases [9,10].

Despite its effectiveness, it is essential to consider that GFD has different nutritional issues: it can cause macronutrient and calorie-intake unbalance and a lack of dietary fibers [11]. Previous studies have found that children on a GFD consumed more significant quantities of fats, proteins, and calories than controls [12]. It is also important to underline that many processed gluten-free foods have a high glycemic index [13]. These factors have been conducted to point out the risk of CeD patients on a GFD to develop metabolic syndrome and metabolic-associated fatty liver disease [14,15,16,17]. Recent guidelines suggest that once the diagnosis of CeD has been confirmed, patients should be referred to a dietitian for dietary instruction on following a GFD, and be advised to follow a balanced diet and a healthy, active lifestyle [18].

Many studies have evaluated the nutritional statuses of CeD patients, mainly focusing on the number of macro-micronutrient assumptions, common nutritional deficiencies, and body composition, using the body mass index [BMI] as the only available parameter; moreover, most of these studies targeted the pediatric population [19,20,21,22,23,24]. Therefore, we aimed to evaluate nutritional status using different nutritional parameters in a cohort of CeD adults at diagnosis and after starting GFD, also comparing them to healthy controls.

## 2. Materials and Methods

### 2.1. Study Design and Participants

This prospective study was conducted at the Department of Surgery, Oncology, and Gastroenterology of the University Hospital of Padua after Ethical Committee Approval (Protocol AOPD1410) in March 2022. The study population included CeD patients enrolled at the moment of diagnosis and followed up for one year on a GFD, and healthy controls, recruited among staff familiars and friends. Written informed content was obtained from all the participants. The inclusion criteria for CeD patients were a CeD diagnosis based on positive anti-transglutaminase IgA antibodies, normal total IgA, and the presence of villous duodenal atrophy, classified as Marsh 3 according to the Marsh classification [25] or based on ESPGHAN guidelines for CeD diagnosis in pediatric patients [26]. We excluded CeD dosing transglutaminase IgA antibodies associated with total IgA dosage for healthy controls. We only included subjects aged 18–65 years at enrolment. Exclusion criteria for all groups were the diagnosis of other inflammatory and/or psychological diseases, previous history of cancer, the presence of a pacemaker or other electric devices, pregnancy, or breastfeeding. We also considered a BMI > 30 kg/m^2^ an exclusion criterion because bioelectrical impedance data, particularly Phase Angle, could be altered in obese subjects due to body cell mass and tissue hydration. Thus, the results of BIA could not reflect their real nutritional status [27].

### 2.2. Anamnesis and Physical Activity Assessment

At the enrolment visit, data regarding CeD diagnosis, particularly clinical presentation and histological and serological tests, were recorded for all CeD patients. Clinical presentations were divided into the classical (anemia, weight loss, or diarrhea taken as indices of malabsorption), non-classical (other signs and symptoms than those with the classical presentation), and asymptomatic presentation (family history of CeD or associated autoimmune diseases) [28].

We collected information about current symptoms and serology during follow-up and evaluated dietary adherence using the Biagi questionnaire [29]. The presence of comorbidities was also reported, with particular attention to other autoimmune disorder diagnoses. Moreover, we collected the participants’ physical activity levels using the validated International Physical Activity Questionnaire-Short Form (IPAQ-SF) [30,31].

### 2.3. Nutritional Status and Body Composition

Data were collected at 3 time points for newly diagnosed CeD patients: at the moment of the diagnosis, and after 6 and 12 months on a GFD; for controls, only one visit took place. All the exams were performed in the morning; participants fasted for at least 4 h and relaxed in the 15 min before.

Body weight and height were measured (to the nearest 0.5 kg and 0.5 cm, respectively) with a professional balance scale (Akern MPE 205K, a100HM, with altimeter) to assess and classify BMI according to the World Health Organization (WHO) guidelines: <18.5 underweight, 18.5–24.9 average weight, 25–29.9 overweight and >30 obese [32].

Anthropometric measurements were taken according to the International Standards for Anthropometric Assessment [33], using a metallic anthropometric tape on the right side of the participants. Furthermore, bioelectrical impedance analysis (BIA) was performed to estimate body composition with 800 µA current, 50 kHz sinusoidal wave (Akern BIA 101, Akern SRL). The measurements were performed in the supine position, following 5 min of rest, with abducted arms (30 degrees) and legs (45 degrees); 4 electrodes were applied, 2 on the dorsal surface of the right hand and 2 on the right feet, 5 cm distant. For each patient, 2 assessments were conducted to provide reliable and reproducible data, following BIA guidelines [34,35].

Values of resistance and reactance given by BIA were analyzed with dedicated software (Bodygram Plus) to obtain data regarding body composition in terms of the fat mass (FM, in kg and %), fat-free Mass, which includes everything but the fat (FFM, kg and %), fat-free mass index (FFMI) and appendicular skeletal mass index (ASMI), these latter two consider lean mass, total and appendicular, adjusted for height. Body cell mass, the metabolically active component of the body (BCM, kg/m) and body cell mass index (BCMI, BCM/height^2^), total body water, and extracellular water, which is composed of out-of-cell fluids (TBW and ECW, liters an %), and the phase angle (PA), the angular transformation of the ratio of reactance (Xc) and resistance (R), calculated with the formula PA = arc tangent/(Xc/R)*(180/π), were all measured. Due to these reasons, PA is influenced by sex, age, and physical health [36]. The assessment of body composition, evaluating these parameters, is a crucial tool to evaluate nutritional status and its changes related to a dietary intervention.

### 2.4. Dietary Habits and Intakes

All the participants were asked to record three-day 24 h diaries, two weekdays, and one weekend day, after being instructed; patients had to report the number of meals to list all the food and beverages consumed during the 3 days, particularly the weight of each product (grams). The diary was completed, for newly diagnosed CeD patients, at the moment of the diagnosis (T0), after 6 months (T1) and 12 months (T2) on a GFD. The analysis of the diaries permitted us to evaluate each participant’s diet, particularly to determine macronutrient intakes. Results were compared to what the Italian Society of Human Nutrition recommends in the LARN (Reference Levels of Nutrients and Energy Intake for the Italian Population).

### 2.5. Data Analysis

Nutritional data recorded with the three-day 24 h diaries were analyzed with METADIETA computer software (version 4.0) to estimate journal energy and macronutrient intakes for CeD patients and controls.

All other data were analyzed with the software STATA11 (Stata Corp., College Station, TX, USA, version 11.2). Possible differences between patients and controls were assessed with the independent-samples *t*-test or Mann–Whitney test for parametric and non-parametric variables. The paired-samples *t*-test or Wilcoxon signed-rank test for parametric and non-parametric variables were used to assess the effects of the GFD in CeD patients on all parameters collected. The significant levels were set at *** *p* < 0.001, ** *p* < 0.01, and * *p* < 0.05.

## 3. Results

A total of 32 newly diagnosed CeD patients were enrolled at the CeD center of the Department of Surgery, Oncology, and Gastroenterology of the University Hospital of Padua during the study period. Of these, 2 patients refused to participate in the study for lack of time, 2 had BMI > 30, and 4 were lost during follow-up. Finally, 24 CeD patients were included in the study. Moreover, 28 sex and age-matched controls were accepted to be included in the study after CeDs were excluded.

### 3.1. Demographic Data and Lifestyle

CeD patients comprised 16 females (67%) with a mean age at diagnosis of 34 ± 13 years. The overall clinical presentation was non-classical (58%). The healthy controls group comprised 17 females (61%) with a mean age at the enrolment of 34 ± 11 years. Data regarding demographic and clinical characteristics, lifestyle, and habits are summarized in Table 1.

**Figure 1:**
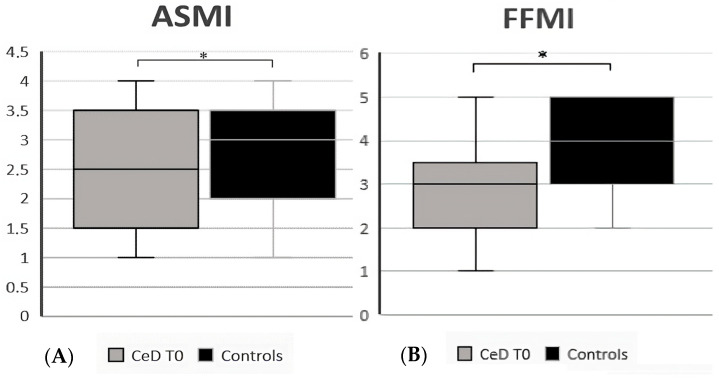
(**A**) ASMI (appendicular skeletal muscle mass index) in CeD patients vs. controls; (**B**) FFMI (fat-free mass index) in CeD patients vs. controls. * Significant differences (*p* < 0.05).

**Figure 2:**
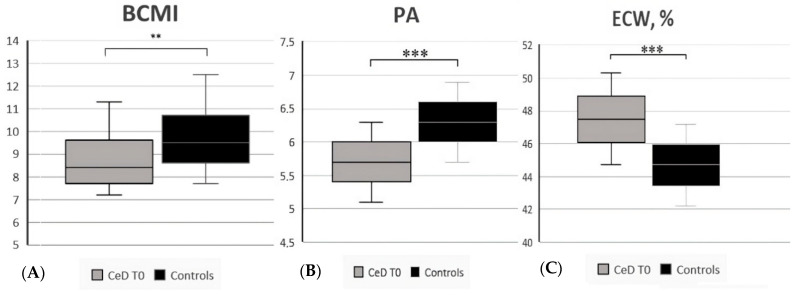
(**A**) BCMI (body cell mass index) in CeD patients vs. controls; (**B**) PA (phase angle) in CeD patients vs. controls; (**C**) ECW (extracellular water, %) CeD patients vs. controls. ** Significant differences (*p* < 0.01), *** (*p* < 0.001).

Comparing CeD patients at diagnosis (T0) with controls, data showed that CeD patients at diagnosis had a lower BCMI, with an average value of 8.4 (7.2–11.3) vs. 9.5 (7.7–12.5) (*p* = 0.006), a lower FFMI [3 (1–5) vs. 4 (2–5), *p* = 0.02], and a lower ASMI [2.5 (1–4) vs. 3 (1–4), *p* = 0.02]. Moreover, the phase angle was significantly lower in CeD patients than in controls, with a mean value of 5.7 ± 0.6 vs. 6.3 ± 0.6 (*p* < 0.001), while the percentage of ECW was higher (47.5 ± 2.8 vs. 44.7 ± 2.5, *p* < 0.001). All data are reported in Table 2, and significant results are reported in Figure 1 and Figure 2.

To evaluate the changes caused by a GFD in CeD patients, we compared the nutritional status at diagnosis with that after 6 months (T1) and 12 months (T2).

We found that the phase angle after six months on a GFD (T1) improved compared to T0, with a mean value of 5.9 ± 0.8 vs. 5.7 ± 0.6 (*p* = 0.04). Similarly, we observed an improvement in the average handgrip value [29 (25.7–36.8) vs. 28.1 (16.5–48.7), *p* = 0.02] and MQI [1.3 (1.2–1.4) vs. 1.2 (0.9–1.8), *p* = 0.02]. Conversely, ECW significantly decreased from T0 to T1 (47.5 ± 2.8 vs. 46.1 ± 3.6, *p* = 0.02), and both BCMI and ECW in liters improved.

**Figure 3:**
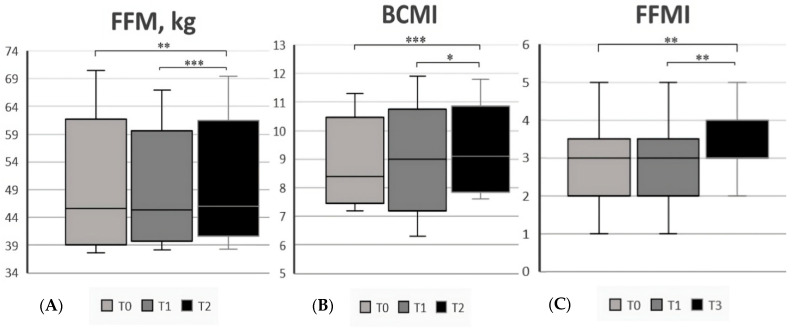
(**A**) FFM (fat-free mass, kg) in CeD patients at the 3 different time points; (**B**) BCMI (body cell mass index) in CeD patients at the 3 different time points; (**C**) FFMI (fat-free mass index) in CeD patients at the 3 different time points. * Significant differences (*p* < 0.05), ** (*p* < 0.01), *** (*p* < 0.001).

**Figure 4:**
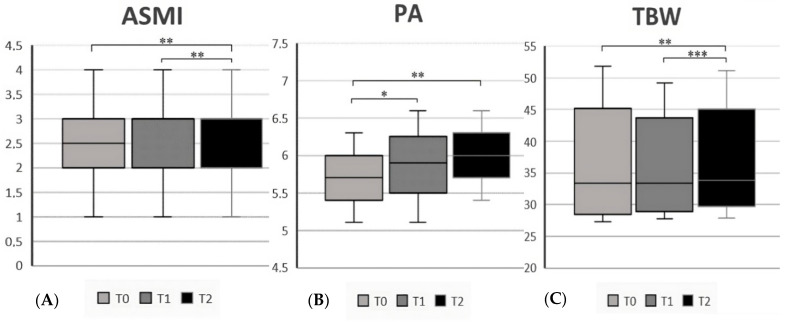
(**A**) ASMI (appendicular skeletal muscle mass index) in CeD patients at the 3 different time points; (**B**) PA (phase angle) in CeD patients at the 3 different time points; (**C**) TBW (total body water) in CeD patients at the 3 different time points. * Significant differences (*p* < 0.05), ** (*p* < 0.01), *** (*p* < 0.001).

**Figure 5:**
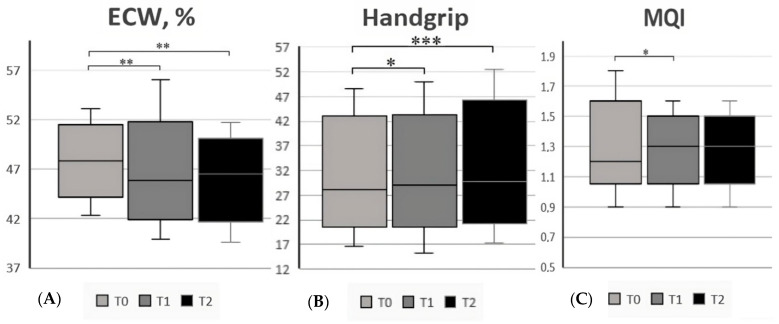
(**A**) ECW (extracellular water, %) in CeD patients at the 3 different time points; (**B**) handgrip value in CeD patients at the 3 different time points; (**C**) MQI (muscle quality index) in CeD patients at the 3 different time points. * Significant differences (*p* < 0.05), ** (*p* < 0.01), *** (*p* < 0.001).

After one year on a GFD (T2), we reported an overall improvement in the nutritional status. As shown in Supplementary Table S1, FFM [46 (38.3–69.4) vs. 45.6 (37.6–70.5), *p* = 0.004], BCMI [9.1 (7.6–11.8) vs. 8.4 (7.2–11.3), *p* = 0.02], FFMI [3 (2–5) vs. 3 (1–5), *p* = 0.01] and ASMI [3 (1–4) vs. 2.5 (1–4), *p* = 0.008] significantly increased over time. Additionally, the mean value of PA (6 ± 0.6 vs. 5.7 ± 0.6, *p* = 0.002) and the average handgrip value increased [29.7 (17.3–52.5) vs. 28.1 (16.5–48.7), *p* < 0.001]. In the end, TBW increased [33.8 (27.9–51.1) vs. 33.4 (27.3–51.8), *p* = 0.004] while the percentage of ECW decreased (47.5 ± 2.8 vs. 45.9 ± 3.1 *p* = 0.01). We also analyzed the changes in all parameters from T1 and T2, finding an improvement in the following: FFM, FFMI, ASMI, BCMI, PA, and TBW. No differences were found in terms of BMI values. All results are reported in Supplementary Table S1. In addition, Figure 3, Figure 4 and Figure 5 report all statistically significant data. We also considered the nutritional parameters in CeD patients at diagnosis based on clinical presentation without finding significant differences between the two groups regarding body composition (Supplementary Table S2).

### 3.2. Dietary Habits and Intakes

**Figure 6:**
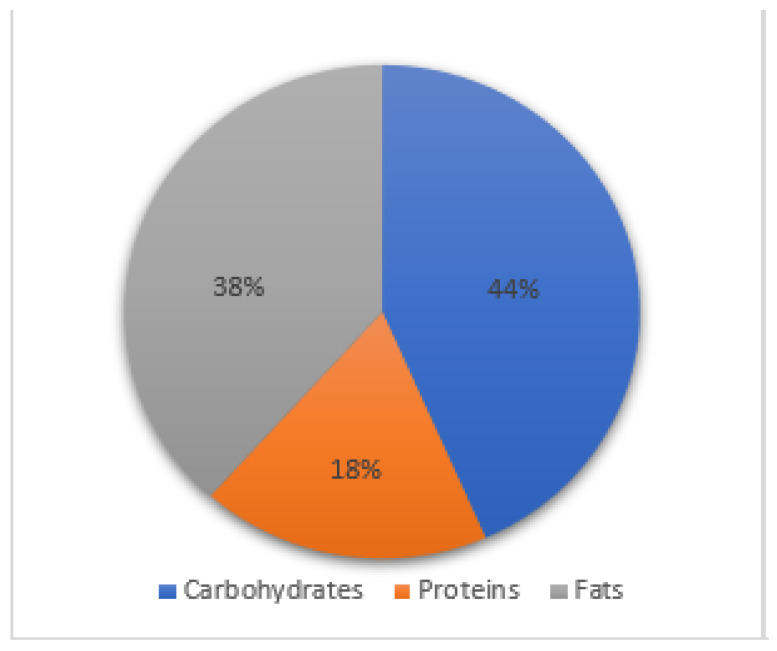
CeD macronutrients daily assumption.

**Figure 7:**
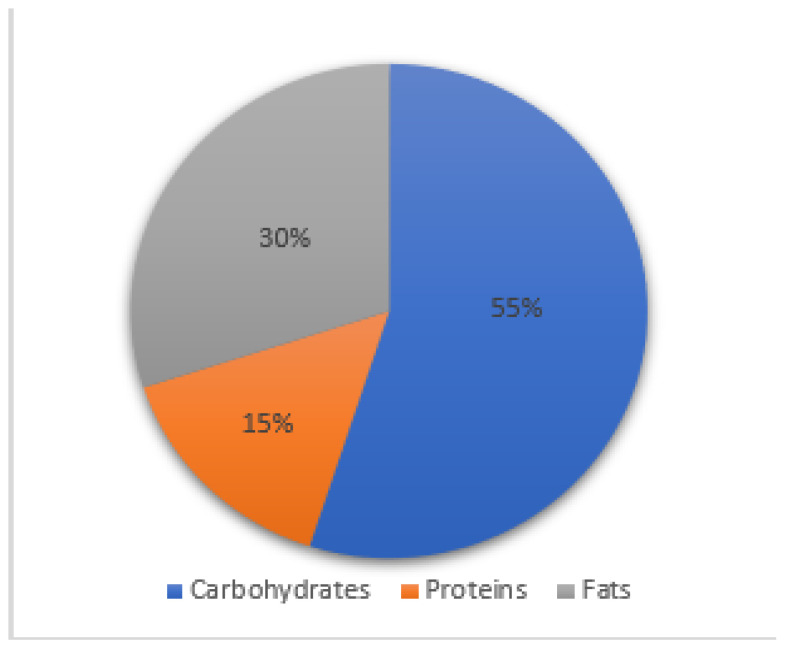
Reference Levels of Nutrients and Energy Intake for the Italian Population.

CeD patients at diagnosis had an imbalanced diet compared to what the Italian Society of Human Nutrition recommends in the LARN. The mean assumption of carbohydrates was 43 ± 7%, 18 ± 3% of proteins, and 38 ± 5% of fats, versus the recommended proportion: 50–55% carbohydrates, 15–20% proteins, and 30% of fats (Figure 6 and Figure 7). In particular, we observed that patients tended to avoid carbohydrates as the font of energy, preferring foods that do not contain grains in their main meals and assuming more fats. The dietary habits did not change at 6 and 12 months on a GFD.

## 4. Discussion

According to recent guidelines, once the diagnosis of CeD is confirmed, patients should be referred to a dietitian for starting a GFD. The effect of this diet on the nutritional statuses of CeD patients is still a matter of debate since many studies have mainly focused on BMI as the only available parameter. Therefore, in the current study, we aimed to evaluate the nutritional status of newly diagnosed CeD patients using different nutritional parameters, including those derived by applying bioelectrical impedance analysis. We observed that CeD patients at diagnosis had a poorer nutritional status than healthy controls, but it significantly improved after 6 and 12 months of a GFD. 

Only a few studies have been performed on adult CeD patients using BIA. In particular, Capristo et al., similarly to us, evaluated the nutritional status in two groups: CeD patients at the moment of diagnosis and CeD patients on a GFD, comparing them to healthy controls [37]; both groups of patients had a lower BMI, FM, and FFM compared to controls, while our data showed only a reduced FFMI at diagnosis. Most nutritional status studies have focused on the pediatric population, as emphasized in a recent meta-analysis and systematic review performed by Vereczkei et al. [38], where five studies of the seven included were conducted on children. This meta-analysis underlined that the few studies conducted on adults, at diagnosis, and during follow-up, had used different techniques to evaluate body composition [38]. In particular, most studies analyzed the nutritional status of CeD patients using only BMI or other anthropometric measures [20,23,39,40]. Our results emphasize that the analysis of those parameters alone does not necessarily show any differences between patients and controls. In contrast, data obtained with BIA allowed us to demonstrate statistically significant differences concerning BCMI, FFMI, ASMI, and PA, which were lower in CeD than in controls, and ECW percentage, which was higher in CeD patients. Thus, CeD patients’ nutritional status at diagnosis is worse than healthy subjects, particularly considering both PA and ECW percentages, known to be predictors of malnutrition. PA also correlates with BCM and ECW ratios. So, it is widely used in clinical practice as a cellular integrity and cell functions index that decreases in cases of inflammation, malnutrition, impaired quality of life, and sarcopenia [41,42,43,44,45]. We also considered the changes in nutritional status over time due to the effect of the GFD. Similarly, in this setting, the studies are few and mainly carried out in the pediatric population [38], or only based on BMI [46,47,48].

Our results showed a body-fluid redistribution on a GFD: TBW increased (*p* < 0.001), as well as PA (*p* = 0.002), while ECW percentage decreased, mainly in the first 6 months of the diet (*p* = 0.004). These data confirm that GFD has a positive effect on nutritional status. Indeed, the TBW increase reflects the rising of all fat-free mass indexes. On the other hand, considering the improvement in PA and the decrease in ECW, which are known to correlate with chronic inflammation, it is possible to speculate that these changes reflect a recovery of inflammation status in CeD patients [49]. No correlation with the clinical presentation was observed in our study.

As described above, we found no change in BMI levels on a GFD compared to those in other studies [44,45,46,47,48]. Ukkola et al. [48] investigated changes in BMI after 1 year of a GFD in 698 CeD patients. They observed that BMI tended to “normalize”, increasing in underweight patients and decreasing in overweight ones. However, this study did not consider BMI changes due to a different caloric intake, and data regarding weight and height were self-reported.

We also evaluated the diet composition of our patients, finding a lower use of carbohydrates and higher consumption of fat compared to the average values. These results align with the available literature focusing on the nutritional imbalance of a GFD. Patients following a GFD have a lower intake of carbohydrates, probably because of the fear of consuming gluten, and a higher consumption of fats, probably because they are used as an alternative to cereals and because of the higher content of fats in GFD products [11,12,13]. These results support the need for dietary counseling when starting a GFD and a periodic follow-up to prevent nutrient deficiencies but mostly to avoid GFD-linked conditions, such as obesity and metabolic alterations, that different authors observed [14,15,16,17,50,51,52,53]. For example, Rispo et al. investigated the incidence of MAFLD and NAFLD in 221 CeD patients at the moment of diagnosis and after 2 years of a GFD. They found a significant increase in both conditions on a GFD [16]. The study of Ciccone et al. also showed a high risk of developing metabolic syndrome and hepatic steatosis in CeD patients on a GFD [15]. It is essential to consider that all parameters detected by BIA are highly variable and influenced by inflammation. Therefore, a long-time follow-up would also be helpful to check whether patients maintain an excellent nutritional profile and a well-controlled inflammation status.

The main strength of our study is that it is one the few that has evaluated CeD patients’ nutritional statuses over time and compared them to healthy controls using BIA, showing the limitation of using only BMI or anthropometric measures. However, this study has some limitations. The most important is the low sample size which could affect some results; the authors believe that this study can be used as a model for future research with a more extensive study population. In addition, we did not evaluate nutritional status regarding micronutrients and serologic markers, so further research is necessary to find possible correlations between body composition and the risk of developing dysmetabolic conditions.

In conclusion, our study demonstrates that CeD patients have a worse nutritional status at diagnosis than healthy subjects, but that this improves on a GFD. We show that nutritional status evaluation is a complex process that cannot only be based on the evaluation of BMI but needs the analysis of specific parameters, with the support of a specialist dietitian, as suggested by the more recent guidelines. Dietary counseling is essential for proper nutritional education, preventing nutrient deficiencies, and avoiding GFD-linked conditions such as metabolic disorders.

## Figures and Tables

**Table 1:** Study population characteristics and habits.

Demographic Characteristics	CeD Patients	Controls	CeD vs. Controls
			*p* (chi-squared test)
Age (mean ± SD)	34 ± 13	34 ± 11	
Females (*n*, %)	16 (67%)	17 (61%)	
CeD familiarity (*n*, %)	5 (21%)	0 (0%)	
Comorbidities (*n*, %)	8 (33%)	7 (25%)	
Lifestyle			
Smoking (*n*, %)			
- Never smoke - Current smokers - Previous smokers	13 (54%) 6 (25%) 5 (21%)	20 (72%) 4 (14%) 4 (14%)	*p* = 0.43
Alcohol consumption (*n*, %)			
- Never - Rarely - Every other day - Everyday	8 (34%) 14 (58%) 1 (4%) 1 (4%)	9 (32%) 18 (64%) 1 (4%) 0 (0%)	*p* = 0.99
Sugary drinks (*n*, %)			
- Never - Rarely - Every other day - Everyday	14 (58%) 9 (38%) 0 (0%) 1 (4%)	14 (50%) 12 (42%) 1 (4%) 1 (4%)	*p* = 0.91
Sugary foods (*n*, %)			
- Never - Rarely - Every other day - Everyday	1 (4%) 6 (25%) 5 (21%) 12 (50%)	0 (0%) 7 (25%) 8 (29%) 13 (46%)	*p* = 0.93
Physical activity levels (*n*, %)			
- Sedentary - Low activity - Moderate activity - Strong activity	12 (50%) 8 (33%) 3 (13%) 1 (4%)	12 (42%) 10 (36%) 5 (18%) 1 (4%)	*p* = 0.94

Nutritional Status and Body Composition.

**Table 2:** Nutritional status parameters in CeD patients compared to healthy controls.

	CeD T0 *n* = 24	Controls *n* = 28	CeD vs. Controls	
			*p* (Mann–Whitney)	*p* (*t*-test)
Fat Mass (kg)	12.9 (5.7–25)	14.6 (5.9–30.3)	0.63	
Fat-Free Mass (kg)	45.6 (37.6–70.5)	49.1 (38.7–64.5)	0.09	
Body Cell Mass Index	8.4 (7.2–11.3)	9.5 (7.7–12.5)	0.006	
Fat Mass (%)	22.1 (9.9–37.1)	21.7 (11.7–34.9)	0.85	
Fat-Free Mass (%)	77.9 (62.9–90.1)	78.3 (65.1–88.3)	0.94	
Fat-Free Mass Index	3 (1–5)	4 (2–5)	0.02	
Appendicular Skeletal Muscle Index	2.5 (1–4)	3 (1–4)	0.02	
Body Mass Index (mean ± SD)	21.9 ± 3.2	22.8 ± 2.5		0.2
Phase Angle (°, mean ± SD)	5.7 ± 0.6	6.3 ± 0.6		<0.001
Total Body Water	33.4 (27.3–51.8)	36 (28.2–47.4)	0.09	
Extracellular Water (L)	16 (12.8–27.7)	16.5 (13.2–20.7)	0.60	
Extra-Cellular Water (%, mean ± SD)	47.5 ± 2.8	44.7 ± 2.5		<0.001
Waist (cm, mean ± SD)	78.7 ± 11.1	77.1 ± 6.6		0.53
Abdominal Fat (%, mean ± SD)	2.9 ± 1.8	2.7 ± 1.1		0.53
Handgrip Value	28.1 (16.5–48.7)	32.5 (20.1–54.8)	0.19	
Muscle Quality Index (mean ± SD)	1.2 (0.9–1.8)	1.2 (0.9–3.3)	0.81	

## Data Availability

Data generated or analyzed during this study are available from the corresponding author upon reasonable request.

## Supplementary Materials

The following supporting information can be downloaded at: https://www.mdpi.com/article/10.3390/nu15122686/s1, Table S1: Nutritional parameters at T0, T1, and T2, and relative comparisons. Table S2: Nutritional status parameters of CeD patients with classical and non-classical presentations.
