# Supplementary material for: Assessment of Nutritional Status by Bioelectrical Impedance in Adult Patients with Celiac Disease: A Prospective Single-Center Study
Source: Nutrients. 2023 Jun 9;15(12):2686. doi: 10.3390/nu15122686 (PMC10303882; doi:10.3390/nu15122686)
Supplement: Supplementary file 1 [file nutrients-15-02686-s001.zip › nutrients-2429715-supplementary.pdf]

Table S1: Nutritional parameters at T0, T1 and T2, and relative comparisons.

	**T0**	**T0 vs T1**			**T0 vs T2**			**T1 vs T2**	
		T1	P (Wilcoxon signed-rank test)	P (t-test)	T2	P (Wilcoxon signed-rank test)	P (t-test)	P (Wilcoxon signed-rank test)	P (t-test)
Fat Mass (kg)	12.9 (5.7-25)	13.9 (5.9-24.2)	0.24		12.9 (5.3-24.3)	0.80		0.10	
Fat Free Mass (kg)	45.6 (37.6-70.5)	45.3 (41.4-52.3)	0.85		46 (38.3-69.4)	**0.004**		**<0.001**	
Body Cell Mass Index	8.4 (7.2-11.3)	9 (8.1-9.6)	0.06		9.1 (7.6-11.8)	**0.02**		**<0.001**	
Fat Mass (%)	22.1 (9.9-37.1)	23.4 (15.7-28.6)	0.42		21.1 (11-36.4)	0.49		0.08	
Fat Free Mass (%)	77.9 (62.9-90.1)	76.6 (71.5-84.3)	0.42		78.9 (63.6-89)	0.48		0.08	
Fat Free Mass Index	3 (1-5)	3 (2-4)	0.53		3 (2-5)	**0.01**		**0.005**	
Appendicular Skeletal Muscle Index	2.5 (1-4)	3 (2-3)	0.65		3 (1-4)	**0.008**		**0.005**	
Body Mass Index (mean±SD)	21.9±3.2	22.1±3		0.31	22.2±3.1		0.15		0.43
Phase Angle (°, mean±SD)	5.7±0.6	5.9±0.8		**0.04**	6±0.6		**0.002**		0.2
Total Body Water	33.4 (27.3-51.8)	33.3 (30-38.2)	0.9		33.8 (27.9-51.1)	**0.004**		**<0.001**	
Extra-Cellular Water (L)	16 (12.8-27.7)	16 (14.2-17.2)	0.07		16 (12.9-23.8)	0.16		0.40	
Extra-Cellular Water (%, mean±SD)	47.5±2.8	46.1±3.6	**0.006**		45.9±3.1	**0.01**		0.95	
Waist (cm, (mean±SD)	78.7±11.1	78.7±9.7	0.95		79.5±9.6	0.32		0.18	
Abdominal Fat (%, mean±SD)	2.9±1.8	2.9±1.6	0.94		3.1±1.6	0.29		0.15	
Handgrip Value	28.1 (16.5-48.7)	29 (25.7-36.8)	**0.02**		29.7 (17.3-52.5)	**<0.001**		0.10	
Muscle Quality Index (mean±SD)	1.2 (0.9-1.8)	1.3 (1.2-1.4)	**0.02**		1.3 (0.9-1.6)	0.08		0.47	

Table S2. Nutritional status parameters of CeD patients with classical and non-classical presentation.

	**Classical Presentation n=10**	**Non-Classical Presentation n=14**		
			P (Wilcoxon signed-rank test)	*P (t-test)*
Fat Mass (kg)	11 (6.7-24.9)	9 (5.7-25)	0.35	
Fat Free Mass (kg)	43.9 (38.4-56.4)	47.1 (37.6-70.5)	0.27	
Body Cell Mass Index	8.4 (7.6-9.6)	8.4 (7.2-11.3)	0.66	
Fat Mass (%)	19.5 (11.6-37.1)	24 (9.9-36.6)	0.73	
Fat Free Mass (%)	80.5 (62.9-88.4)	76 (63.7-90.1)	0.73	
Fat Free Mass Index	3 (1-4)	3 (2-5)	0.42	
Appendicular Skeletal Muscle Index	2 (1-3)	3 (1-4)	0.42	
Body Mass Index (mean±SD)	21±3.1	22.5±3.1		0.25
Phase Angle (°, mean±SD)	5.7±0.5	5.6±0.6	0.60	
Total Body Water	32.2 (27.9-41.4)	34.5 (27.3-51.8)	0.27	
Extra-Cellular Water (L)	15.4 (12.8-19.7)	16.7 (13.3-27.7)	0.07	
Extra-Cellular Water (%, mean±SD)	47.1±2.7	47.7±3		0.60
Waist (cm, (mean±SD)	76.2±8.6	80.4±12.5		0.37
Abdominal Fat (%, mean±SD)	2.8±2	3±1.7		0.78
Handgrip Value	28 (20-40)	28.8 (16.5-48.7)	0.81	
Muscle Quality Index (mean±SD)	1.2 (1.1-1.8)	1.3 (0.9-1.4)	0.78	
